# Preventing primary liver cancer: the HBV vaccination project in the Gambia (West Africa)

**DOI:** 10.1186/1476-069X-10-S1-S6

**Published:** 2011-04-05

**Authors:** Ruggero Montesano

**Affiliations:** 1 Former Staff member of the International Agency for Research on Cancer (WHO), Lyon, France. Present address: via dei Giardini 24, 11013 Courmayeur, Italy

## Abstract

The Gambia Hepatitis Intervention Study (GHIS) consisted in the progressive introduction of HBV plasma-derived vaccine in different zones of this African country during the period 1986-1990. The study was launched and coordinated by IARC and is one of the most effective examples of an intervention project that both substantially contributed to our knowledge and to the health of local populations. Similar intervention studies have been carried out in South-East Asia. The studies indicate that the natural history of HBV infection differs in different populations , having a direct relevance for the implementation of HBV vaccination programmes in various parts of the world.

## Article

Recent estimations provided by GloboCa 2008 [1] indicate that in 2008 12.7 million new cancer cases and 7.6 million cancer deaths occurred in the world and that most of these cancers are present in developing countries either in terms of incidence (56%) or mortality (63%). It is also expected that by 2030 some 21.4 million new cancer cases will be diagnosed and over 13.2 million cancer deaths will occur annually. It is estimated that liver cancer (mostly hepatocellular carcinoma [HCC]) is responsible for more that 600,000 cancer deaths worldwide and represents the third most frequent cause of cancer deaths [2,3]. The major causes of hepatocellular carcinoma have been known since several decades and in the developing countries these are HBV, HCV infections and exposure to aflatoxins through the diet [4-6]. Since the early 1980s an efficient and safe vaccine against HBV infection has been available and various intervention studies, namely in Quidong Province, China [7], Taiwan [see 8], and The Gambia [9], have been initiated to assess the efficacy of HBV vaccination at infancy in the prevention of HCC.

All the children vaccinated within the Gambia Hepatitis Intervention Study (GHIS) were registered, resulting in two cohorts of approximately 60,000 children, one of which received only the routine EPI vaccination and the other the HBV vaccine in addition. To determine the response to HBV vaccine and the persistence of vaccine-induced immunity, some 1000 children were recruited consecutively and these have been followed annually to assess their HBV serological status. A cross-sectional survey was carried out at the ages of 4 and 9 years of a similar number of unvaccinated children to determine the vaccine efficacy against HBV carriage status and infection.

The vaccine efficacy shows an 84% protection against infection and 94% against HBV chronic carriage at 9 years of age (See Table 1). The evaluation of the expected protection against the development of HCC is expected from the year 2017 [10]. Similar intervention studies have been carried out in South-East Asia see [7,11]. It is of interest to note that, in the Chinese population, perinatal transmission from mothers positive for HBVe antigen is frequent, whereas in Africa horizontal transmission (sibling-to-sibling) is prevalent. The study in Taiwan shows that the incidence rate of HCC in children of 6-19 years of age was considerably lower in children born after the initiation of the HBV vaccination and that this decrease was not present in children born from mothers who were positive for HBsAg and HBeAg [11]. These studies in Africa and South-East Asia indicate that the natural history of HBV infection differs in these populations and they have a direct relevance in the implementation of HBVvaccination strategy.

**Table 1:** Hepatitis B vaccination trials

Location No. of subjects	Year of recruitment/ year of Follow up	Prevalence HBsAg Non-vaccinated/ vaccinated population	Efficacy	Relative Risk of developing HCC Non-vaccinated/ vaccinated population
The Gambia* >60000	1986-1990/ 9 yrs	10% > 1%	94%	Expected 2017
China* (Qidong)>38000	1984-1990/ 11 yrs	7,1% > 1.66%	75%	Expected >2015
**Taiwan**^♦^	1984	9.8% in 1984 > 0.7% in 1999	84%	0.31^■^

*HBV vaccination trials programme [16,7]

^♦^Universal vaccination programme [11]

^■^64 HCC among vaccinees in 37709304 person-years versus 444 HCC in unvaccinated subjects in 78496406 person-years [11]

It has been estimated that, in a surviving birth cohort for the year 2000, routine infant HBV vaccination, with a 90% coverage and the first dose administered at birth, would prevent 84% of global HBV related deaths, i.e. 1.4 million [12].

In The Gambia the high incidence of HCC is also associated with exposure to the carcinogen aflatoxin B_1_ through the consumption of groundnuts and maize contaminated with the fungi *Aspergillus flavus* and *parasiticum*[6]. Comprehensive studies have been carried out in this country to assess the relative contribution and the interaction of the different risk factors, namely HBV infection and aflatoxin B_1_, in the etiopathogenesis of HCC [**13**,**14**]. These case-control studies, using serological markers of exposure to HBV and aflatoxin B_1_ (as determined by the presence of aflatoxin-albumin adduct and of 249^ser^ p53 mutation in sera DNA), clearly show that exposure to aflatoxins affects the entire population and that exposure to both HBV infection and aflatoxins results in a prominent increase risk of developing HCC.

In summary, these studies show that HBV vaccination programmes have been successfully implemented in various parts of the world. In addition, molecular epidemiological studies in The Gambia and in South-East Asia have clearly shown the relevance of other major risk factors, namely aflatoxin exposure [see 15], in the etiopathogenesis of HCC.

## Competing interests

The author declare that has no competing financial or non-financial interest.

## References

[B1] Ferlay J Shin HR Bray F Forman D Mathers C Parkin DM GLOBOCAN 2008, Cancer Incidence and Mortality Worldwide: IARC CancerBase No. 10 [Internet] 2010 International Agency for Research on Cancer: Lyon, France

[B2] Parkin DM Sitas F Chirenje M Stein L Abratt R Wabinga H Part I: Cancer in indigenous Africans – burden, distribution, and trends Lancet Oncology 2008 9 683 692 10.1016/S1470-2045(08)70175-X 18598933

[B3] Sitas F Parkin DM Chirenje M Stein L Abratt R Wabinga H Part II: Cancer in indigenous Africans – causes and control Lancet Oncology 2008 9 786 795 10.1016/S1470-2045(08)70198-0 18672214

[B4] International Agency for Research on Cancer IARC Monographs on the Evaluation of Carcinogenic Risks to Humans Hepatitis Viruses 1994 59 Lyon: International Agency for Research on Cancer 45

[B5] International Agency for Research on Cancer IARC Monographs on the Evaluation of Carcinogenic Risks to Humans Hepatitis Viruses 1994 59 Lyon: International Agency for Research on Cancer 165

[B6] International Agency for Research on Cancer IARC Monographs on the Evaluation of Carcinogenic Risks to Humans Some Traditional Herbal Medicines, Some Mycotoxins, Naphthalene and Styrene 2002 82 Lyon: International Agency for Research on Cancer 171 PMC4781602 12687954

[B7] Sun Z Ming L Zhu X Lu J Prevention and control of hepatitis B in China J Med Virol 2002 67 447 450 10.1002/jmv.10094 12116043

[B8] Beasly RP Hwang LY Lin CC Chien CS Hepatocellular carcinoma and HBV: a prospective study of 22,707 men in Taiwan The Lancet 1981 2 1129 1133 10.1016/S0140-6736(81)90585-7 6118576

[B9] GHIS The Gambia Hepatitis Intervention Study. The Gambia Hepatitis Study Group Cancer Res 1987 47 5782 5787 2822233

[B10] Viviani S Carrieri P Bah E Hall AJ Kirk GD Mendy M Montesano R Plymoth A Sam O Van der Sande M Whittle H Hainaut P The Gambia Hepatitis Intervention Study 20 years into the Gambia Hepatitis Intervention Study: assessment of initial hypothesis and prospects for evaluation of protective effectiveness against liver cancer Cancer Epidemiol Biomarkers Prev 2008 17 3216 3223 10.1158/1055-9965.EPI-08-0303 18990765

[B11] Chang M-H You S-L Chen C-J Liu C-J Lee C-M Lin S-M Chu H-C Wu T-C Yang S-S Kuo H-S Chen D-S Taiwan Hepatoma Study Group Decreased incidence of hepatocellular carcinoma in hepatitis B vaccinees: a 20-year follow-up study JNCI 2009 101 1348 1355 19759364 10.1093/jnci/djp288

[B12] Goldstein ST Zhou F Hadler SC Bell BP Mast EE Margolis HS A mathematical model to estimate global hepatitis B disease burden and vaccination impact Int J Epidemiol 2005 34 1329 1339 10.1093/ije/dyi206 16249217

[B13] Kirk GD Bah E Montesano R Molecular epidemiology of human liver cancer: insights into etiology, pathogenesis and prevention from The Gambia, West Africa Carcinogenesis 2006 27 2070 2082 10.1093/carcin/bgl060 16679307

[B14] Kirk GD Lesi OA Mendy M Szymañska K Whittle H Goedert JJ Hainaut P Montesano R 249(ser) TP53 mutation in plasma DNA, hepatitis B viral infection, and risk of hepatocellular carcinoma Oncogene 2005 24 5858 5867 10.1038/sj.onc.1208732 16007211

[B15] Wild CP Gong YY Mycotoxins and human disease:a largely ignored global health issue Carcinogenesis 2009 31 71 82 10.1093/carcin/bgp264 19875698 PMC2802673

[B16] Viviani S Jack A Hall AJ Maine N Mendy M Montesano R Whittle HC Hepatitis B vaccination in infancy in The Gambia: protection against carriage at 9 years of age Vaccine 1999 17 2946 2950 10.1016/S0264-410X(99)00178-4 10462228

